# Representing Representation: Integration between the Temporal Lobe and the Posterior Cingulate Influences the Content and Form of Spontaneous Thought

**DOI:** 10.1371/journal.pone.0152272

**Published:** 2016-04-05

**Authors:** Jonathan Smallwood, Theodoros Karapanagiotidis, Florence Ruby, Barbara Medea, Irene de Caso, Mahiko Konishi, Hao-Ting Wang, Glyn Hallam, Daniel S. Margulies, Elizabeth Jefferies

**Affiliations:** 1 Department of Psychology / York Neuroimaging Centre, University of York, Hesslington, York, United Kingdom; 2 Department of Psychology, Sapienza University of Rome, Rome, Italy; 3 Neuroanatomy and connectivity group, Max Planck Institute for Human and Cognitive Brain Sciences, Leipzig, Germany; University of Modena and Reggio Emilia, ITALY

## Abstract

When not engaged in the moment, we often spontaneously represent people, places and events that are not present in the environment. Although this capacity has been linked to the default mode network (DMN), it remains unclear how interactions between the nodes of this network give rise to particular mental experiences during spontaneous thought. One hypothesis is that the core of the DMN integrates information from medial and lateral temporal lobe memory systems, which represent different aspects of knowledge. Individual differences in the connectivity between temporal lobe regions and the default mode network core would then predict differences in the content and form of people’s spontaneous thoughts. This study tested this hypothesis by examining the relationship between seed-based functional connectivity and the contents of spontaneous thought recorded in a laboratory study several days later. Variations in connectivity from both medial and lateral temporal lobe regions was associated with different patterns of spontaneous thought and these effects converged on an overlapping region in the posterior cingulate cortex. We propose that the posterior core of the DMN acts as a representational hub that integrates information represented in medial and lateral temporal lobe and this process is important in determining the content and form of spontaneous thought.

## Introduction

Many of our thoughts and feelings concern goals and events involving people and places absent from the here and now[1, 2]. This capacity reflects activity in a large-scale brain system known as the default mode network [3–5], which is active when people generate thoughts unrelated to the unfolding external world [6–13]. However, we still know little about how different patterns of thought emerge from interactions between components of the DMN.

The functional architecture of the DMN places important constraints on accounts of the biological basis of spontaneous thought. Studies have shown that medial prefrontal and posterior cingulate regions form the ‘core’ of the DMN that integrates information from sub-systems on medial and lateral regions of the temporal lobe [14, 15]. Since these temporal lobe regions represent different aspects of our long-term knowledge of the world, a key function of the core DMN may be to integrate information activated in distinct memory systems. The hippocampus in the medial temporal lobe supports episodic knowledge characterized by specific events from the past [16], spatial navigation [17] and predictions about the future [18]. It has been suggested that this constellation of phenomena reflects the contribution of the hippocampus to multimodal scene construction [19]. Anterior and lateral temporal regions, by contrast, are implicated in the representation of multimodal semantic information that is acquired across many different experiences [20, 21]. The semantic representations formed in these regions allow us to understand the significance of words and objects that are encountered and also to understand more nuanced meanings that emerge from the combination of concepts, and that are potentially highly relevant to evolving patterns of thought. Temporal pole, in particular, has been implicated in the process by which meanings are combined to form a ‘narrative’ and in understanding the emotional connotations of objects, words, and social agents (for a review see [22].

It is hypothesised that the capacity for the core of the DMN to integrate information from anterior and medial aspects of the temporal lobe allows diverse forms of spontaneous thought to be supported in a flexible manner, a view known as the component process account [23]. To test this perspective, resting state functional magnetic resonance imaging was recorded in 87 individuals and, on a subsequent day, experience sampling was used to measure the content of spontaneous thought during a task conducive to mind-wandering. We explored whether individual differences in the functional architecture of the DMN predicted the nature of spontaneous thoughts reported in the later laboratory session, testing two assumptions of the component process account of this networks function. First, if core regions of the DMN allow different representational forms to be integrated, connectivity from the medial, anterior and lateral temporal lobes that vary with experience should overlap in the medial core of the DMN. Secondly, if different types of spontaneous thought can be explained in terms of the integration of information from distinct memory systems, then connectivity from different temporal lobe sites should reflect different types of spontaneous thought.

## Methods

### Design

The current study explores cross-sectional differences between functional connectivity and experience. Neurocognitive function during the resting state was measured on Day One and subjective experience on a subsequent laboratory session on Day Two. This design choice was motivated by limitations in the method for assessing subjective experience. Prior studies have either assessed experience while measuring neural activity online [7–10, 24] or have relied on retrospective measures of the thoughts that emerge during the resting state [14, 25]. Although online experience sampling provides information on the link between experience and on-going neural function, the method disrupts the normal time course of brain activity signals [26]. Moreover, acquiring experience sampling data simultaneously while recording neuroimaging data runs the risk of confounding measures of spontaneous thought with the participants’ expectation of the purpose of the experiment—a problem known as reactivity [26]. Alternative approaches rely on participants’ ability to retrospectively assess the contents of their experience, usually via a questionnaire at the end of the study [25, 27]. Although retrospective measures preserve the natural dynamics of the brain, and minimise the reactive effects of thought probes, they rely on the participant’s ability to correctly remember the experiences they had during the period in question. This method, therefore, raises the concern that problems with memory will lead to biases in the results produced [2]. By assessing the contents of thought using online experience sampling on a different day from the collection of resting state data, we were able to explore individual differences in the covariance between the content of thought and associated neural processes without relying on the participants’ memory. This approach has been successfully used to explore a number of forms of higher-order thought including meta-cognition [28], binocular rivalry [29] and reading comprehension [30].

### Participants

Participants were recruited by advert from the Department of Psychology at the University of York (age range 18–31). They were offered either a payment of £20 or a commensurate amount of course credits. All participants provided written informed consent prior to completing the experiment. The Ethics committee of the York Neuroimaging Centre approved this study, including the process for gaining informed consent. The participants were recruited in two cohorts (Sample 1; n = 39, Sample 2; n = 48) although there were no differences between them germane to this study. To demonstrate the generalizability of the components identified through our decomposition of the experience sampling data we recruited a sample of 67 undergraduate students (age range 18–24) who participated in the same experience sampling paradigm as was used for the resting state experiment. Finally, we used an independent data set to provide independent confirmation of patterns of resting state connectivity identified in this study. These data were from a publicly available data set: the Nathan Kline Institute (NKI)/Rockland Enhanced Sample and contained 141 subjects. Full details of this sample can be found in Gorgolewski et al. [25].

### Behavioural Methods

#### Choice Reaction Time Task

To acquire information about the content of spontaneous thought in a situation conducive to the mind-wandering state, participants performed a simple non-demanding choice reaction time. This task is routinely used in studies of spontaneous thought because it creates periods when spontaneous thoughts are generated as often as during a passive state [31]. Participants sat in a testing booth and were asked to make a parity judgement to numerals that were coloured red. These stimuli were presented in a stream of non-coloured numerals, to which no response was required. Stimuli were presented with a slow inter-stimulus interval (2200–4400 milliseconds) and were on screen for 1000 milliseconds. On average a target occurred 1/6 of the time. Participants responded with the mouse button. Accuracy was high (Mean = .93; SD = .08) and median response time was 895 milliseconds (SD = 163).

Participants performed the choice reaction time task for twenty minutes last in a testing session that lasted approximately one hour and twenty minutes. Other tasks measured aspects of episodic processing (Sample 1) or semantic processing (Sample 2). The relationship of these other tasks to the resting state data will be reported elsewhere.

#### Multi-Dimensional Experience Sampling (MDES)

Spontaneous thought is variable in both form and content, and studies have identified meaningful patterns of covariance through their associations with independent neurocognitive measures. It is thus necessary to quantify this heterogeneity in order to test the hypothesis that different mental experiences depend on different patterns of functional integration between the temporal lobe and the core of the DMN. At unpredictable moments while performing the laboratory task, participants were interrupted and asked to rate different aspects of their experience. Participants were asked to focus their answers on the contents of their experience in the moment immediately prior to the interruption, thereby reducing demands on memory. They responded using a continuous Likert scale. The specific questions that we used are described in Table 1: they were selected from prior studies and examined the content of thoughts (e.g., temporal content relating to the past or future, referent of thought (self or other) and emotional valence) as well as the form these thoughts took (modality–i.e., whether the thoughts were in words or images, the level of detail and intrusiveness etc…).

**Table 1:** Experience sampling questions used in this experiment.

Dimension	Question	Left	Right
**Task**	My thoughts were focused on the task I was performing	Not at all	Completely
**Future**	My thoughts involved future events	Not at all	Completely
**Past**	My thoughts involved past events	Not at all	Completely
**Self**	My thoughts involved myself	Not at all	Completely
**Other**	My thoughts involved other people	Not at all	Completely
**Emotion**	The content of my thoughts was	Negative	Positive
**Images**	My thoughts were in the form of images	Not at all	Completely
**Words**	My thoughts were in the form of words	Not at all	Completely
**Intrusive**	My thoughts were intrusive	Not at all	Completely
**Detail**	My thoughts were vague and non-specific	Not at all	Completely

Whenever experience sampling occurred the questions were administered in a quasi-random order. The first question was always about task focus, followed by blocks of questions about the content and form of thoughts. On each occasion, the order of each mini block, as well as the order of questions within each mini block, was randomized. Participants were probed an average of 8 times during the twenty minute task. We used a fully randomized sequence of experience sampling probes to ensure that regularities in our probing schedule did not bias the results of our experiment [32]. As in previous studies the data from all individuals were concatenated into a single matrix. Prior to decomposition, we z-scored the experience sampling data for each sample separately to minimize the differences between the different samples. The z-scoring was performed on each matrix column separately. We then used PCA with varimax rotation to determine the patterns of covariance in the experience sampling data. In our decompositions we opted for a three-component solution so as to be consistent with prior studies [33–35].

### Neuroimaging Methods

#### Resting state acquisition

Data for both samples were acquired using an eight-channel phased array head coil (GE) tuned to 127.4 mhz on a GE 3 Tesla Signa Excite hdx MRI scanner at York Neuroimaging Centre, at the University of York. Blood oxygen level-dependent (BOLD) contrast images with fat saturation were acquired using a gradient single-shot echo planar imaging (EPI) sequence. The acquisition parameters for Sample 1 were the following: scan duration 7 min, repetition time (TR) 2000 ms, echo time (TE) minimum full (~19 ms), 210 volumes, flip angle 90°, matrix 64 x 64, field of view (FOV) 192 mm, slice thickness 3 mm with a 0.5mm gap and 32 slices with an interleaved (bottom up) acquisition order. For Sample 2 the following parameters were used: scan duration 9 min, repetition time (TR) 3000 ms, echo time (TE) minimum full (~19 ms), 180 volumes, flip angle 90°, matrix 64 x 64, field of view (FOV) 192 mm, slice thickness 3 mm and 60 slices with an interleaved (bottom up) acquisition order.

The functional data were co-registered onto high-resolution structural images. For this reason, a sagittal isotropic 3D fast spoiled gradient-recalled echo (3D FSPGR) structural T1 weighted scan was acquired for each of the participants (TR 7.8 ms, TE minimum full, flip angle 20°, matrix size 256 x 256 x 176, voxel size 1.13 x 1.13 x 1 mm). To facilitate the co-registrations, a high-resolution T1-weighted in-plane anatomical image was also acquired for all participants, using a fluid attenuated inversion recovery (FLAIR).

An independent data set was obtained from the Nathan Kline Institute (NKI) / Rockland Enhanced Sample, a publicly available data set. This allowed us to test the connectivity patterns produced in our present analyses in an independent data set. For the present purposes we used a sample containing 141 subjects that have been previously been used by Gorgolewski et al. [25] and Davey et al., [36]. The resting state fMRI data were acquired with the following parameters: TR 2500 ms, TE 30 ms, 120 volumes, matrix size 72 × 72, 38 slices, flip angle 80°, 0.3 mm spacing between slices, voxel size 3 × 3 × 3 mm and an interleaved slice acquisition order. A high-resolution anatomical image was also acquired for each subject using the MPRAGE sequence.

#### Resting state pre-processing

All fMRI pre-processing and analyses were performed using FSL. We extracted the brain from the skull using the BET toolbox for both the FLAIR and the structural T1 weighted images and these scans were registered to standard space using FLIRT [37]. Prior to conducting the functional connectivity analysis the following pre-statistics processing was applied to the resting state data; motion correction using MCFLIRT [38]; slice-timing correction using Fourier-space time-series phase shifting; non-brain removal using BET [39]; spatial smoothing using a Gaussian kernel of FWHM 6mm; grand-mean intensity normalisation of the entire 4D dataset by a single multiplicative factor; high pass temporal filtering (Gaussian-weighted least-squares straight line fitting, with sigma = 100 s); Gaussian low pass temporal filtering, with sigma = 2.8 s.

#### First level analysis

Seed regions were placed in sites previously identified as being part of the DMN [14]. This study was selected to provide seed locations since it focussed on the relationship between the core DMN and ‘subsystems’ in medial and more anterior parts of the temporal lobe corresponding to different aspects of long-term memory. We included two seeds representing the core of the DMN, in medial pre-frontal cortex (mPFC, MNI -6, 52, -2) and posterior cingulate cortex (pCC, -8, -56, 26). To investigate the integration of information from long-term memory, we also selected three temporal lobe seeds likely to contribute to spontaneous thought in distinctive ways [14]. Two regions were chosen in the anterior temporal lobe–the temporal pole (T POLE, -50, 14, -14) and lateral temporal cortex (LTC, -60, -24, -18). The Hippocampal formation was selected from the medial temporal lobe (HF+, -22, -20, -26). Consistent with Andrews-Hanna and colleagues [14] we only used seeds in the left hemisphere. Full details of all spatial maps are available through Neurovault: http://neurovault.org/collections/1238/.

Our spherical seed rois had a 3mm radius, and were centred on these seed co-ordinates in pCC, mPFC, LTC, TPOLE and the HF+. The time series of these regions were extracted and used as explanatory variables in a separate subject level functional connectivity analysis for each seed. In these analyses, we also included 11 nuisance regressors: the top five principal components extracted from white matter (WM) and cerebrospinal fluid (CSF) masks in accordance with the compcor method [40] and six motion parameters. The WM and CSF masks were generated by segmenting each individual’s high-resolution structural image (using FAST in FSL). The default tissue probability maps, referred to as Prior Probability Maps (PPM), were registered to each individual’s high-resolution structural image (T1 space) and the overlap between these PPM and the corresponding CSF and WM maps was identified. Finally, these maps were thresholded (40% for the SCF and 66% for the WM), binarized and combined. The six motion parameters were calculated in the motion-correction step during pre-processing. Movement in each of the three Cartesian directions (x, y, z) and rotational movement around three axes (pitch, yaw, roll) were included for each individual. No global signal regression was performed [41].

## Results

Our experimental question depends on identifying the shared variance between individual differences in functional connectivity within the DMN subsystems and in the qualities of spontaneous thought. Accordingly there are three steps to our analysis: 1) The determination of different categories of experience during spontaneous thought, 2) the quantification of the functional connectivity of regions of the DMN and 3) the identification of the shared variance between categories of spontaneous thought and the connectivity maps of the DMN.

### Determining Different Types of Spontaneous Thought Using MDES

**Fig 1:**
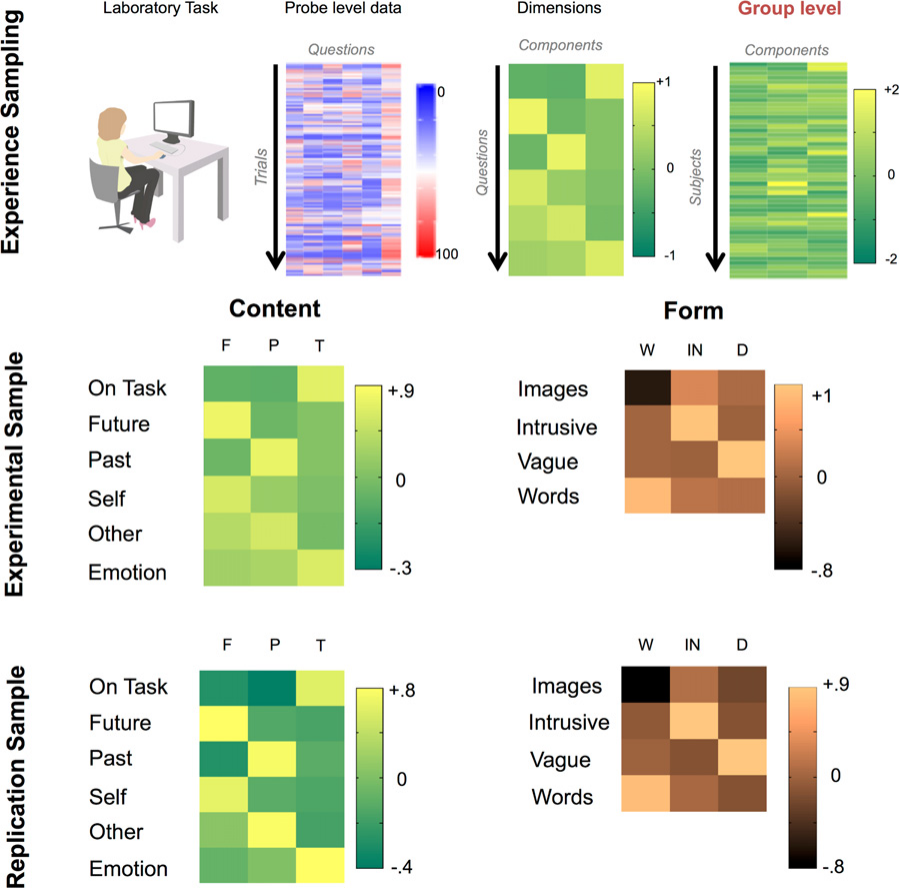
Determining different types of spontaneous thought. The upper panel describes the steps involved in calculating the underlying dimensions that make up the trial level experience sampling data. The middle and lower panel illustrate the result of the application of this decomposition process to both the experimental and replication samples. These heat maps present the loadings of the different questions on the three largest components. Varimax rotation was applied to both data sets. Consistent with prior work, decomposition of the content of experience revealed three orthogonal factors: (1) Future and self-related thoughts (2) Past and other related thoughts and (3) Thoughts which are related to the task and have a positive emotional valence. The decomposition that the form of thoughts took revealed three factors: (1) Thoughts that were either in the form of images or words, (2) Thoughts that were intrusive and (3) Thoughts that were vague and lacked detail. Acronyms: F–Future, P–Past, T–Task, W–Words / Images, IN–Intrusive, D–Detail.

We assessed the content of spontaneous thought using multiple questions and decomposed these using principal components analysis (PCA) allowing patterns of covariance that broadly correspond to different types of thought to be identified. The pipeline for the decomposition of experience sampling data is explained schematically in the upper panel of Fig 1.

We decomposed reports of the content of the experience and the form in separate analyses [33, 34]. For the decomposition of content we focused on questions relating to temporal focus, referent of thought, task focus and emotional content. Consistent with prior investigations we found three orthogonal factors (See Lower Left hand Panel of Fig 1): i) *Future- and self-focused thoughts*: individuals with a high weighting on this component were often thinking about themselves in the future, accounting for 29% of the observed variance, ii) *Past-focused social thoughts*: individuals with a high weighting on this component were often thinking about self and others in the past, accounting for 19% of the variance and iii) *Task-related thoughts*: individuals with a high weighting on this component were often thinking about the task itself and experienced fewer negatively-valanced off-task thoughts, accounting for 18% of the variance. A low weighting on this latter component may be of relevance to the DMN given this system is important in task unrelated thinking [13]. These components are presented visually in the middle left hand panel of Fig 1.

Next, the questions regarding the form of thoughts–such as whether these were experienced as images or words, whether they were detailed and whether they intrusive–were decomposed in a similar way. This yielded three components which can be seen in the middle right hand panel of Fig 1: 1) *The modality of the thoughts* (Images or Words): individuals with a high weighting often described their thoughts as containing words rather than images and this reflected 33% of the variance, 2) *The level of intrusiveness of the thoughts*: individuals with a high weighting often described their thoughts as intrusive, accounting for 26% of the variance and 3) *The level of detail* in the thoughts: individuals with a low weighting on this reported more detail in their thoughts accounting for 23% of the overall variance.

To assess the stability of these solutions we repeated the decomposition on an independent data set (see Behavioural Methods for further details) and found very similar results (see Fig 1 lower panel). Different patterns of thoughts uncovered using this MDES method are therefore stable across different samples from the same population.

### Determining the Connectivity of Core and Temporal Regions of the DMN

**Fig 2:**
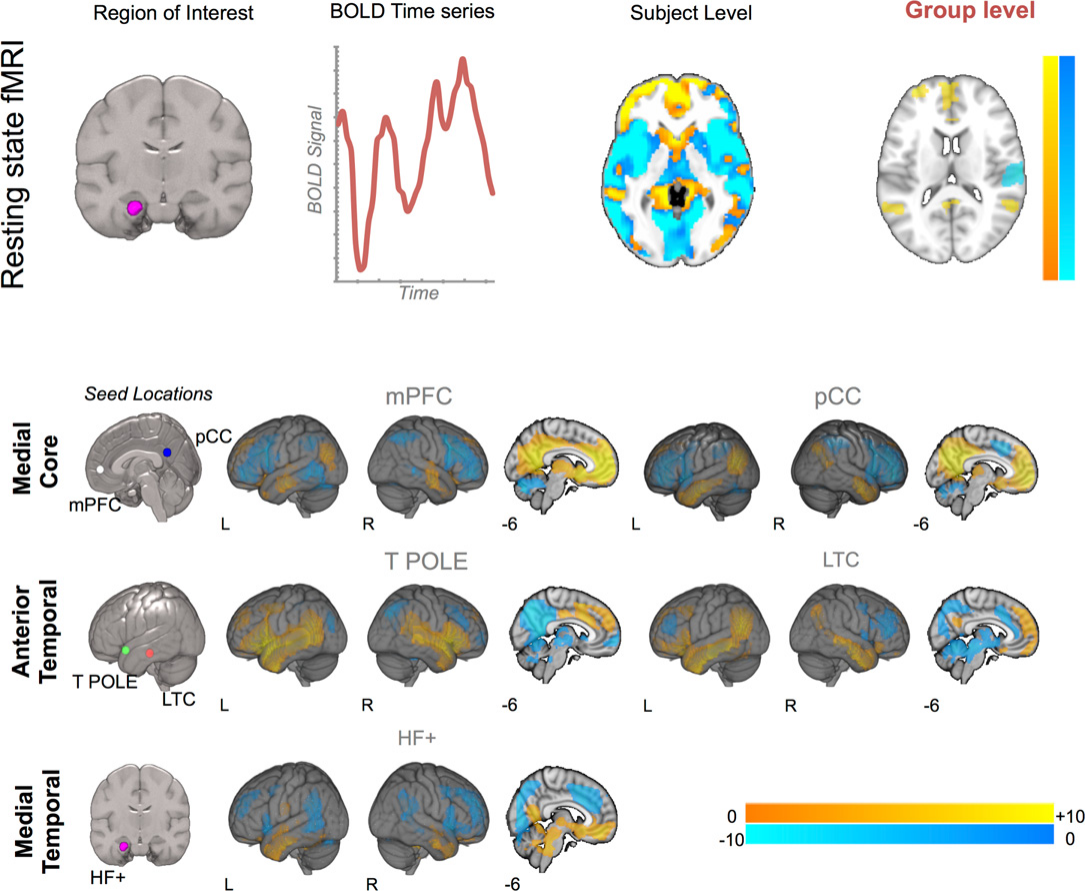
Determining the connectivity of core and temporal lobe regions of the Default Mode Network. The upper panel presents the analysis pipeline employed to generate the functional connectivity maps for each of the five seed regions included in this analysis. The lower panel presents the results of a seed-based connectivity analysis from each of the seed regions. The location of the seeds is shown in the left most image in each row. Spatial maps were thresholded at Z < 2.3 and corrected at p < .05. Acronyms: mPFC–medial pre-frontal cortex, pCC–Posterior Cingulate Cortex, T POLE–temporal pole, LTC—lateral temporal cortex, HF+–Hippocampal Formation.

The next analysis identifies the functional connectivity profiles of different regions of the DMN. We used the five seed regions to drive whole-brain functional connectivity analysis using the pipeline schematized in the upper panel of Fig 2 (see *Resting State Methods* for further details). This analysis produced group-level maps for each seed region and these are presented in the main panel of Fig 2. It can be seen that these regions show connectivity patterns consistent with the DMN. One notable exception is the connectivity map of the temporal pole: Although this region is highly coupled to other regions of the lateral temporal lobe and dorsal regions of the mPFC, it shows patterns of reduced correlation with the anterior mPFC and the pCC. This pattern of differential connectivity is broadly consistent with the component process framework suggested by Andrews-Hanna and colleagues [14].

### Describing the Shared Cross-Sectional Variance between Different Patterns of Spontaneous Thought and the Functional Connectivity of the DMN

Having documented the different qualities of experience reported during spontaneous thought, as well as the functional connectivity of the selected seed regions, we next explored their shared variance. We projected the components identified by MDES back into the subject space by averaging the component loadings at the level of each individual. We conducted five separate group level multiple regressions in FSL using FMRIB's Local Analysis of Mixed Effects (FLAME). We used automatic outlier detection to minimize the consequence of extreme scores in both brain and mental experience. Connectivity maps for a particular region of the DMN were the dependent variable. Each model included individual averages in each dimension of spontaneous thought as between-participant explanatory variables. Scan type was included as a nuisance covariate, modelling scanning sequence as a binary regressor. We examined whole brain differences in the positive and negative connectivity of each seed region with the rest of the brain that could be accounted for by different forms of experience. Since prior work has already established the similarities and differences between prospective and retrospective spontaneous thoughts (e.g., [31, 33–35]), we also examined the positive and negative correlations related to the similarities between prospective and retrospective thoughts (i.e. A positive weighting on both Future and Past) and their differences (i.e. Future thinking greater than Past and the reverse). We set a cluster forming threshold of Z = 2.3 and controlled for Type I errors by controlling for (i) the number of voxels in the brain, (ii) the two-tailed nature of our contrasts and (iii) the five models. This yielded an alpha value of p < .005 FWE.

**Fig 3:**
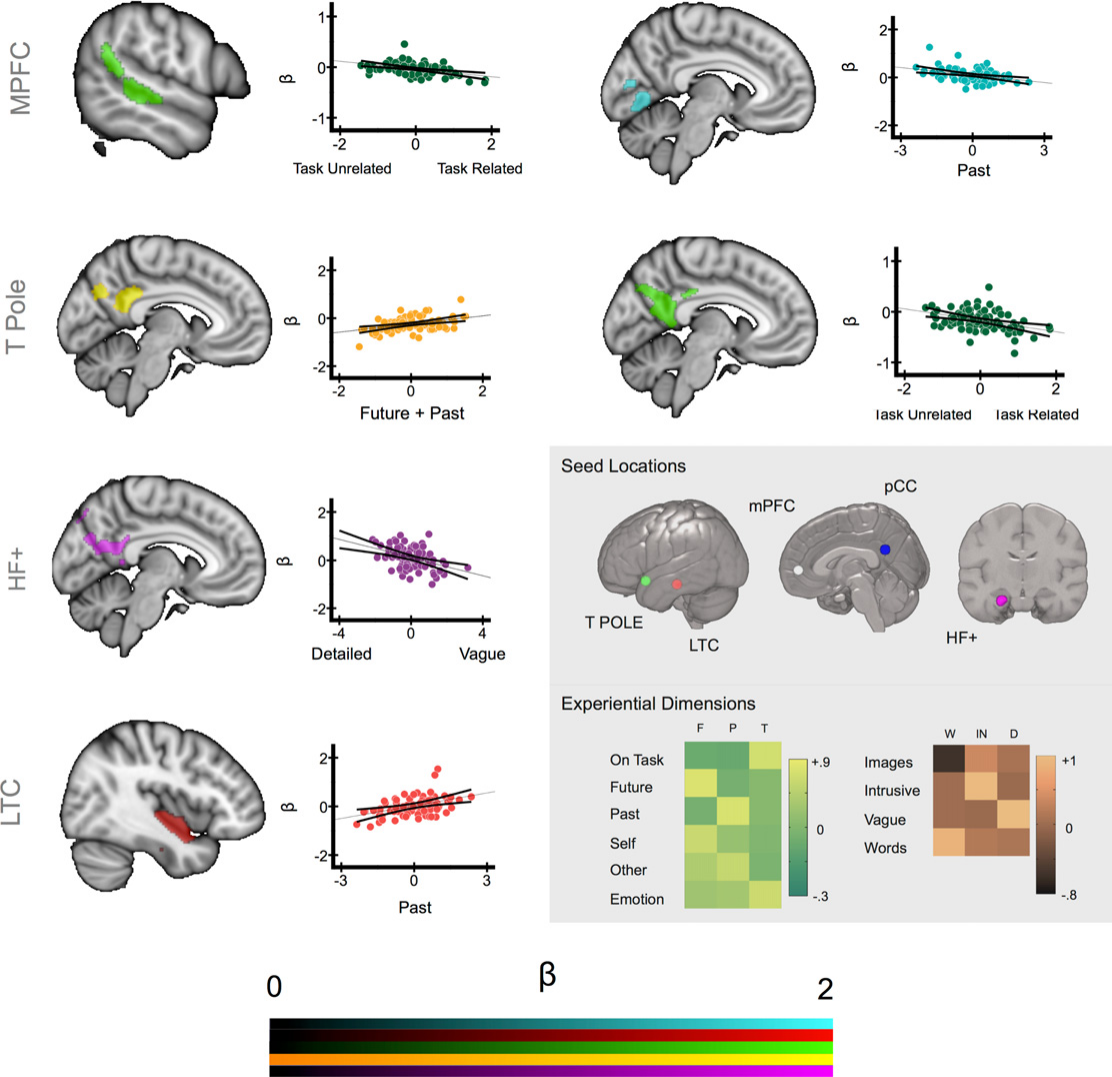
Different types of spontaneous thoughts vary with the functional connectivity of different aspects of the DMN. Cluster corrected maps illustrating the aspects of the functional connectivity maps of the seed regions that share co-variance with different dimensions of experience. The scatterplots illustrate the average beta values extracted from these regions and plotted against the residual scores for each dimension for each participant. The grey sub-panel illustrates the seed regions and experiential regressors upon which these maps were based. Spatial maps were thresholded at Z < 2.3 and corrected for multiple comparisons at p < .005 (FWE). Acronyms: mPFC–medial pre-frontal cortex, T POLE–temporal pole, LTC—lateral temporal cortex, HF+–Hippocampal Formation. F–Future, P–Past, T–Task, W–Words / Images, IN–Intrusive, D–Detail.

These analyses identified six contrasts explaining significant amounts of the variance in the individual level connectivity maps that were associated with cross-sectional differences in the MDES scores. These clusters are presented in Fig 3 and reflect regions whose connectivity with the seed region showed a pattern of covariance with a particular category of experience across our sample. The location and spatial extent of these clusters are described in Table 2. For each of these clusters we extracted the average parameter estimates from within the cluster corrected maps for each individual and plotted these against the weightings of each individual for each type of experience generated using MDES. These data are presented in the scatter plots in Fig 3. The data for all of these models is available on line in Neurovault at the following URL: http://neurovault.org/collections/1238/.

**Table 2:** Clusters showing a significant association between a dimension of experience and functional connectivity at rest. The p-values represent the level of significance after correcting for the number of voxels in the brain. Contrasts marked with an asterisk identify regions that are significant after correcting for multiple comparisons.

**Seed**	**MNI Coordinates (x,y,z)**	**MDES Dimension**	**Cluster Centre of Gravity**	**Cluster Size (voxels)**	**Region**	**P—Value**
mPFC	-6 52–2	Future +	-41 14–16	613	Left Anterior IFG / Anterior Insula	0.04
		Future—Past	1–71–1	1708	Medial Occipital Cortex	0.0001*
		Past -	0–74 2	1359	Medial Occipital Cortex	0.0006*
		Task -	56–26 1	1083	Right Superior Temporal Gyrus	0.002*
		Task -	-56–39 7	683	Right Superior Temporal Gyrus	0.03
		Future + Past +	-39 9–9	872	Left Anterior IFG / Anterior Insula	0.009
pCC	-8–56 26	Past +	58–50 14	594	Right Inferior Parietal Lobule	0.05
HA	-22-20–26	Vague -	-1–60 24	911	Posterior Cingulate Cortex	0.005*
T POLE	-50 14–14	Future +	1–57 20	1386	Posterior Cingulate Cortex	0.0007*
		Intrusive +	46–8–9	614	Right Superior Temporal Gyrus	0.05
		Task -	1–54 23	1814	Posterior Cingulate Cortex	<0.0001*
		Future + Past +	1–54 25	1190	Posterior Cingulate Cortex	0.0018*
LTC	-60–24–18	Past +	-1–48–1	1716	Right Superior Temporal Gyrus	<0.0001*
		Past +	42–3–12	831	Bi-lateral Retrosplenial Cortex	0.009
		Vague -	46 20 36	647	Right Dorsolateral Pre-frontal Cortex	0.03
		Future + Past +	-2–63 17	861	Ventromedial Prefrontal Cortex	0.008
		Future + Past +	42–5–8	710	Right Superior Temporal Gyrus	0.02
		Future + Past +	-1 48–15	681	Posterior Cingulate Cortex	0.02

Connectivity of the temporal pole seed yielded overlapping clusters in posterior cingulate cortex that showed a positive correlation with mental time travel (Future + Past) and a negative correlation with the extent to which thoughts were positive and focused on the task. This suggests that greater coupling between the temporal pole and the posterior cingulate cortex is independently predictive of increased mental time travel (a form of thought involving different temporal perspectives and different social agents) and negative task unrelated thoughts. Seeding from the lateral temporal cortex revealed a cluster in the right hippocampus whose connectivity with the seed was greater for individuals who tended to think about the past more during spontaneous thought. Finally, seeding from the hippocampal formation showed greater functional connectivity with a region of posterior cingulate cortex for individuals whose thoughts were detailed/specific rather than vague.

The regressions from seeds in the core regions of the DMN (i.e. mPFC and pCC) revealed associations with experience for medial prefrontal but not posterior cingulate regions. There was a negative correlation between increasing levels of task focus and the degree of coupling between mPFC and a region of the right superior temporal gyrus. There was also a negative correlation between mPFC and an area of medial occipital cortex for thoughts about the past, with an overlapping response for the contrast Past–Future (see Table 2 for the description of this cluster). Thus, this region in medial occipital cortex was more functionally coupled to mPFC for future more than past thinkers, a pattern that could reflect the process of perceptual decoupling that is hypothesised to be important in the mind-wandering state (see Discussion).

**Fig 4:**
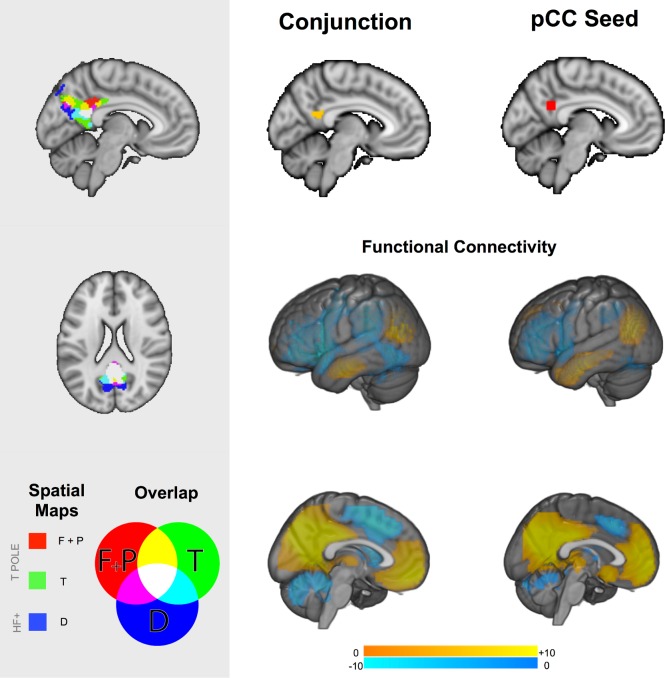
The posterior cingulate cortex is a hub integrating different forms of representational information. The left-hand panel illustrates statistical overlap in the association between three connectivity patterns associated and different types of spontaneous thoughts in the posterior cingulate cortex. The right-hand panel shows the similarity between the formal conjunction of these regions with the seed used in our analysis in terms of spatial location (top) and functional connectivity (bottom). Spatial maps in the main panel were thresholded at Z < 2.3 and corrected for multiple comparisons at p < .05 (FWE). Acronyms: T POLE–temporal pole, HF+–Hippocampal Formation.

Consistent with the importance of the medial core of the DMN in integrating information from medial and temporal regions, visual inspection of Fig 3 indicates that three of the six contrasts identified in our analysis were focused on a similar region of the posterior cingulate cortex. Consistent with the role of pCC as a convergence zone, greater mental time travel was associated with enhanced connectivity from the LTC seed to the same region of the posterior cingulate cortex (p < .02 whole brain corrected). Although this comparison does not pass our threshold for the number of seeds that we selected it does lend support to the role of the pCC as a convergence zone. To formally quantify the spatial commonality of these different maps we overlapped the cluster corrected maps from each contrast. This analysis is presented in the left hand panel of Fig 4 in which it can be seen that these different maps converge on the same region on the posterior segment of the medial surface. To identify the spatial location of this overlap we conducted a formal conjunction of these three spatial maps and the resulting image is presented in the middle panel of Fig 4 (Centre of mass: 1, -54, 20, Cluster Size: 285 voxels). It can be seen that this region is spatially consistent with the seed region that reflects the focus of the posterior core of the DMN as hypothesised by Andrews-Hanna and colleagues (see lower panel).

Studies in both humans and macaques have demonstrated that posterior cingulate regions are known to have a complex heterogeneous pattern of connectivity [42–44] so we performed a further analysis to identify whether our overlap region had a different functional connectivity profile to the seed region in pCC used in our analyses. We used the conjunction cluster generated by the prior analytic step as a mask to drive a functional connectivity analysis in an independent data set (see Resting-State methods). The resulting map is compared to the functional connectivity of the pCC seed region from the current experiment in Fig 4: This demonstrates that these regions show highly similar connectivity. This analysis confirms that association between spontaneous thought and the connectivity of the lateral and medial aspects of the temporal lobe converge on a region that is both anatomically and functionally overlapping with a region commonly accepted to reflect the posterior core of the DMN.

**Fig 5:**
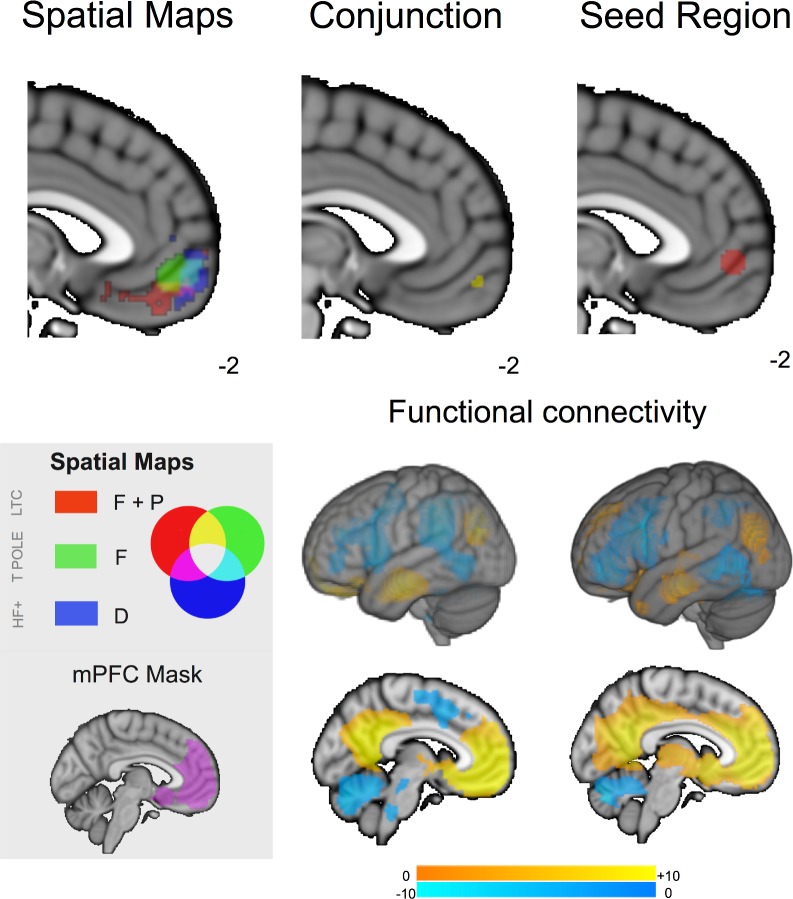
Region of interest analysis exploring the integration of information from the medial and anterior temporal lobe in the medial prefrontal cortex. Analysis restricted to the mPFC indicated that three experience related connectivity patterns converged on the mPFC (left Top Panel). Although a spatial conjunction did reveal a pattern of overlap that was ventral to the mPFC seed region from Andrews-Hanna and colleagues (2010) this was smaller than for the pCC. The lower panel displays the spatial map generated by seeding the region identified through the spatial conjunction in comparison with the map generated by the seed region in the mPFC. Spatial maps were thresholded at Z < 2.3 and corrected for multiple comparisons at p < .05 (FWE). Acronyms: T POLE–temporal pole, LTC—lateral temporal cortex, HF+–Hippocampal Formation.

Finally, although our analysis highlighted the pCC as an important zone of convergence for patterns of connectivity associated with different features of experience, the absence of this pattern in the mPFC may be a result of our conservative analytic strategy that was designed to minimize Type 1 error. To address this concern we generated a mask of the mPFC based on the connectivity to this region from the pCC in our data (Cluster size = 7114 voxels, cluster centre -2, 44, 10, see Fig 5 left hand panel) and repeated the multiple regressions for the LTC, TPOLE and HF+ seeds using this mask to constrain the search space. These more liberal analyses indicated three experience related patterns of connectivity that converged on the mPFC: (i) Greater connectivity from the TPOLE was associated with greater levels of future thought, (ii) Greater connectivity from the LTC was associated with greater mental time travel and (iii) Greater connectivity from the HF+ was associated with more detailed thoughts (see Table 3). A formal conjunction of these regions yielded a small region of convergence (Fig 5, middle panel, Centre of Gravity, 1, -57, -10, Cluster Size = 32 voxels) that was ventral to the mPFC seed from [14] (see Fig 5, Left Hand Panel). We seeded this region in an independent data set (See Resting-state methods) and compared the pattern of connectivity from this region with the seed region from the mPFC (see Fig 5, bottom right hand panel). It is clear that this spatial map was broadly similar to that derived from seeding the medial prefrontal cortex seed in our initial analysis.

**Table 3:** Clusters showing a significant association between a dimension of experience and functional connectivity at rest when using an inclusive mask of the mPFC (see Fig 5). The p-values represent the level of significance after correcting for the number within the mask.

**Seed**	**MNI Coordinates (x,y,z)**	**MDES Dimension**	**Cluster Centre of Gravity**	**Cluster Size (voxels)**	**P—Value**
HF+	-22-20–26	Vague -		520	0.005
T POLE	-50 14–14	SF +		438	0.01
		SF + P -			
LTC	-60–24–18	F+ P +		579	<0.005

## Discussion

Our results are compatible with the hypothesis that the medial core of the DMN is important in spontaneous cognition through its capacity to integrate information from different long-term memory systems located in the medial and anterior regions of the temporal lobe [3,14]. We identified statistically robust patterns of experience that were stable across different samples and demonstrated that individual differences in the strength of these patterns related to variability in DMN connectivity assessed using resting-state fMRI. A key observation was that associations between experience and patterns of connectivity from different regions of the temporal lobe converged on the posterior core of the DMN, in the pCC. This pattern is consistent with the capacity of the pCC to echo functional activity from many different networks [44] as well its status as a member of a ‘rich club’ of influential hub regions that impact on information flow throughout the cortex [45, 46]. In functional terms, a recent meta-analytic decomposition of the posterior cingulate linked this region of cortex to cognitive processes associated with memory and emotion [47], which are important in spontaneous thought [26]. Similarly a meta analytic comparison of mind-wandering and episodic thinking highlighted this region of pCC as common to both [7]. The notion of pCC as a representational hub for spontaneous thought gains further support from its role in other states of complex cognition such as creativity [48] and why this region may be important in meditative states [49, 50]. Building on this convergence of structural, functional, and psychological evidence, our data suggests that variance in spontaneous thought relies on the posterior core of the DMN for the integration of multiple sources of information allowing this region to contribute to different types of thought in a flexible manner [23].

We also found evidence that the medial and lateral regions of the temporal lobe differentially co-operate with the core to shape the content and form of spontaneous cognition. Connectivity of the temporal poles with the pCC was predictive of both greater mental time travel involving social agents and unpleasant task-unrelated thoughts. Studies have shown that a large proportion of off-task thought takes the form of mental time travel [51–53] and unhappiness is a common correlate of off task thought [1, 54, 55]. The convergence of connectivity from the temporal pole to the posterior core of the DMN for these cardinal dimensions of spontaneous thought suggests this region plays an important role in several well-established features of experience, compatible with its previously-recognised role in the retrieval of social and emotionally-laden conceptual information [22]. Connectivity from the medial temporal lobe also shared variance with forms of spontaneous thought. Heightened connectivity from the hippocampus to the posterior cingulate cortex predicted greater specificity to thought–a finding that is a consistent with a role of both HF+ and pCC in generating details within an imaginary scene [56]. Together these patterns of connectivity support the hypothesised role of the DMN subsystems in contributing to different qualities of spontaneous thought [14].

Our results also suggest a difference in emphasis in the roles the posterior and anterior regions of the core of the DMN play in spontaneous thought. Connectivity associated with variation in mental experience converged on the posterior core of the DMN, suggesting this region acts as a representational hub, whereas evidence that the mPFC acts as a convergence zone was only observed with a liberal analytic approach. Nevertheless, connectivity *from* the mPFC seed to posterior regions in superior temporal and visual cortex was associated with variation in patterns of thought. Increased connectivity from mPFC was observed for individuals whose thoughts were focused away from the task (in pSTG) and reduced connectivity for people who thought about the past more than the future (in visual cortex). In our study, reduced connectivity from mPFC to visual cortex might reflect perceptual decoupling–i.e., reduced attention to the external visual world, when attention is directed towards self-generated cognitive states [27, 57, 58]. This might play a particular role in protecting past episodic thought, which involves scene construction, from interference from the external scene (for a discussion of decoupling see [59, 60]). Consistent with a heightened role of scene construction in past related thought, we observed that past thinking was associated with enhanced connectivity between the left LTC seed and the right hippocampus (see Fig 3). In addition, off-task thinking might be promoted by enhanced connectivity with right pSTG extending dorsally into the parietal cortex. A recent data-driven parcelation of the right temporo-parietal region found this region involved two antagonistic patterns of connectivity—a posterior cluster associated with internal states and an anterior cluster relating to more externally focused states [61]. Bzdok and colleagues argue that this antagonism could reflect a role of the rTPJ in switching between internal and external domains. Notably the dorsal extension of the cluster from our data falls between these two clusters, a pattern that may indicate a role for the mPFC in co-ordinating switches in attentional state.

These interpretations linking the mPFC to decoupling and attentional switching suggest that the anterior DMN core could be important in the process through which the underlying elements of cognition are co-ordinated in the service of the on-going train of thought. Consistent with this interpretation, anterior medial prefrontal cortex is activated by information with high personal or emotional significance (e.g. [62]) suggesting that it may represent the importance of inputs to the individual. More generally, the medial prefrontal cortex supports distinct networks for meta-cognition across different task domains [28, 63–65] indicating that it could adjust thought and behaviour on a momentary basis in line with an individual’s goals. Regardless of these specific interpretations, the observed pattern of mPFC connectivity suggests that it may play a role that extends beyond the integration of information during spontaneous thought.

Although these data demonstrate that integration within the posterior core of the DMN is important for different types of spontaneous thoughts, there are a number of important caveats that must be taken into account when considering these data. Our analysis links individual differences in functional connectivity analyses with the content and form of spontaneous thought recorded on a subsequent day, a strategy that guarantees a relatively pure account of the neural dynamics at rest at the cost of detailed information on the momentary correlates of different experiential states. Consequently the neural patterns that predict cross-sectional differences in spontaneous thought may not be identical to those that support these states on a momentary basis (for further discussion of this issue see [8]). More generally, spontaneous thoughts are themselves an emergent property of different cognitive components such as episodic memory and emotional processing [60], and so our data reflect an association between emergent properties at the experiential level (e.g. Mental time travel) and neural level (e.g. Connectivity with the pCC). Our study shows that a distributed neural network is critical for understanding the content and form of spontaneous thought–but it does not directly constrain the psychological interpretations of the neurocognitive operations implicated in spontaneous thought. Finally, it is also important to recognise that we are yet to fully understand the extent to which states of mind-wandering are a stable trait, or are instead a more state like phenomena. Our experiential and neural measurements were recorded within days of each other and so associations between them could reflect the temporary state the participants were in, or could potentially index a stable trait of the participants. It is also unclear the extent to which the content of thoughts we have identified in the short laboratory session correspond to the thoughts that emerge in daily life. In the future a more protracted sampling of participants thoughts as they move through their lives is warranted. It is important to note that it is not necessary to assume that the content of thought recorded in the laboratory correspond to the experience that occurred in the resting state scan for our data to be informative on the mechanisms through which spontaneous thoughts arise: Almost all known psychological states are assumed to vary across participants and this cross-sectional variation is a valid way in which to understand the underlying mechanisms that govern the phenomena.

In conclusion, our results highlight the posterior region of the core of the DMN as an integrator of information from both medial and anterior aspects of the temporal lobe that is important in spontaneous thought. Connectivity patterns associated with three different aspects of experience overlapped in this region, compatible with the suggestion that pCC is receiving information from several systems involved in memory representation and that differences in the strength of these connections give rise to distinct spontaneous mental experiences reflecting the availability of different forms of informational codes. Our data also suggest that the balance of integration from temporal pole and medial temporal regions reflects the form that thoughts take, consistent with studies showing that these different regions represent different aspects of our knowledge of the world. Together these data support an account of the DMN in which the medial core, and in particular the posterior cingulate cortex, functions as a representational hub, enabling it to integrate information from different cortical regions that provide the basis of the experiential content to which we attend when we transcend the here and now using imagination.

## References

[1] Killingsworth MA, Gilbert DT. A wandering mind is an unhappy mind. Science. 2010;330(6006):932–. 10.1126/science.1192439 21071660

[2] Smallwood J, Schooler JW. The restless mind. Psychological bulletin. 2006;132(6):946 17073528 10.1037/0033-2909.132.6.946

[3] Andrews‐Hanna JR, Smallwood J, Spreng RN. The default network and self‐generated thought: component processes, dynamic control, and clinical relevance. Annals of the New York Academy of Sciences. 2014. 10.1111/nyas.12360 PMC4039623 24502540

[4] Buckner RL, Andrews-Hanna JR, Schacter DL. The brain's default network: anatomy, function, and relevance to disease. Annals of the New York Academy of Sciences. 2008;1124:1–38. Epub 2008/04/11. 10.1196/annals.1440.011 . 18400922

[5] Raichle ME, MacLeod AM, Snyder AZ, Powers WJ, Gusnard DA, Shulman GL. A default mode of brain function. Proc Natl Acad Sci U S A. 2001;98(2):676–82. Epub 2001/02/24. 10.1073/pnas.98.2.676 11209064 PMC14647

[6] Fox KC, Spreng RN, Ellamil M, Andrews-Hanna JR, Christoff K. The wandering brain: Meta-analysis of functional neuroimaging studies of mind-wandering and related spontaneous thought processes. NeuroImage. 2015;111:611–21. 10.1016/j.neuroimage.2015.02.039 25725466

[7] Stawarczyk D, D'Argembeau A. Neural correlates of personal goal processing during episodic future thinking and mind‐wandering: An ALE meta‐analysis. Human brain mapping. 2015. 10.1002/hbm.22818 PMC6869624 25931002

[8] Kucyi A, Davis KD. Dynamic functional connectivity of the default mode network tracks daydreaming. Neuroimage. 2014;100:471–80. 10.1016/j.neuroimage.2014.06.044 24973603

[9] Christoff K, Gordon AM, Smallwood J, Smith R, Schooler JW. Experience sampling during fMRI reveals default network and executive system contributions to mind wandering. Proceedings of the National Academy of Sciences. 2009;106(21):8719–24. 10.1073/pnas.0900234106 PMC2689035 19433790

[10] Allen M, Smallwood J, Christensen J, Gramm D, Rasmussen B, Jensen CG, et al The balanced mind: the variability of task-unrelated thoughts predicts error monitoring. Frontiers in human neuroscience. 2013;7. 10.3389/fnhum.2013.00743 PMC3819597 24223545

[11] Tusche A, Smallwood J, Bernhardt BC, Singer T. Classifying the wandering mind: Revealing the affective content of thoughts during task-free rest periods. NeuroImage. 2014 Epub 2014/04/08. 10.1016/j.neuroimage.2014.03.076 . 24705200

[12] Mason MF, Norton MI, Van Horn JD, Wegner DM, Grafton ST, Macrae CN. Wandering minds: the default network and stimulus-independent thought. Science. 2007;315(5810):393–5. 17234951 10.1126/science.1131295 PMC1821121

[13] Stawarczyk D, Majerus S, Maquet P, D’Argembeau A. Neural correlates of ongoing conscious experience: both task-unrelatedness and stimulus-independence are related to default network activity. PLoS One. 2011;6(2):e16997–e. 10.1371/journal.pone.0016997 21347270 PMC3038939

[14] Andrews-Hanna JR, Reidler JS, Sepulcre J, Poulin R, Buckner RL. Functional-anatomic fractionation of the brain's default network. Neuron. 2010;65(4):550–62. 10.1016/j.neuron.2010.02.005 20188659 PMC2848443

[15] Yeo BT, Krienen FM, Sepulcre J, Sabuncu MR, Lashkari D, Hollinshead M, et al The organization of the human cerebral cortex estimated by intrinsic functional connectivity. Journal of neurophysiology. 2011;106(3):1125–65. 10.1152/jn.00338.2011 21653723 PMC3174820

[16] Aggleton JP, Brown MW. Episodic memory, amnesia, and the hippocampal–anterior thalamic axis. Behavioral and brain sciences. 1999;22(03):425–44. 11301518

[17] Epstein RA. Parahippocampal and retrosplenial contributions to human spatial navigation. Trends in cognitive sciences. 2008;12(10):388–96. 10.1016/j.tics.2008.07.004 18760955 PMC2858632

[18] Schacter DL, Addis DR, Buckner RL. Remembering the past to imagine the future: the prospective brain. Nature Reviews Neuroscience. 2007;8(9):657–61. 17700624 10.1038/nrn2213

[19] Hassabis D, Maguire EA. Deconstructing episodic memory with construction. Trends in cognitive sciences. 2007;11(7):299–306. 17548229 10.1016/j.tics.2007.05.001

[20] Visser M, Jefferies E, Lambon Ralph MA. Semantic processing in the anterior temporal lobes: a meta-analysis of the functional neuroimaging literature. Journal of cognitive neuroscience. 2010;22(6):1083–94. Epub 2009/07/09. 10.1162/jocn.2009.21309 . 19583477

[21] Binney RJ, Embleton KV, Jefferies E, Parker GJ, Lambon-Ralph MA. The ventral and inferolateral aspects of the anterior temporal lobe are crucial in semantic memory: evidence from a novel direct comparison of distortion-corrected fMRI, rTMS, and semantic dementia. Cerebral Cortex. 2010;20(11):2728–38. 10.1093/cercor/bhq019 20190005

[22] Olson IR, Plotzker A, Ezzyat Y. The enigmatic temporal pole: a review of findings on social and emotional processing. Brain. 2007;130(7):1718–31. 17392317 10.1093/brain/awm052

[23] Andrews‐Hanna JR, Smallwood J, Spreng RN. The default network and self‐generated thought: component processes, dynamic control, and clinical relevance. Annals of the New York Academy of Sciences. 2014;1316(1):29–52. 24502540 10.1111/nyas.12360 PMC4039623

[24] Smallwood J, Andrews-Hanna J. Not all minds that wander are lost: the importance of a balanced perspective on the mind-wandering state. Frontiers in psychology. 2013;4:441 Epub 2013/08/24. 10.3389/fpsyg.2013.00441 23966961 PMC3744871

[25] Gorgolewski KJ, Lurie D, Urchs S, Kipping JA, Craddock RC, Milham MP, et al A correspondence between individual differences in the brain's intrinsic functional architecture and the content and form of self-generated thoughts. PloS one. 2014;9(5):e97176 10.1371/journal.pone.0097176 24824880 PMC4019564

[26] Smallwood J, Schooler JW. The science of mind wandering: empirically navigating the stream of consciousness. Annual review of psychology. 2015;66:487–518. 10.1146/annurev-psych-010814-015331 25293689

[27] Barron E, Riby LM, Greer J, Smallwood J. Absorbed in thought the effect of mind wandering on the processing of relevant and irrelevant events. Psychol Sci. 2011. 10.1177/0956797611404083 21460338

[28] Baird B, Smallwood J, Gorgolewski KJ, Margulies DS. Medial and lateral networks in anterior prefrontal cortex support metacognitive ability for memory and perception. The Journal of Neuroscience. 2013;33(42):16657–65. 10.1523/JNEUROSCI.0786-13.2013 24133268 PMC6618531

[29] Baker DH, Karapanagiotidis T, Coggan DD, Wailes-Newson K, Smallwood J. Brain networks underlying bistable perception. NeuroImage. 2015;119:229–34. 10.1016/j.neuroimage.2015.06.053 26123379

[30] Smallwood J, Gorgolewski KJ, Golchert J, Ruby FJ, Engen H, Baird B, et al The default modes of reading: modulation of posterior cingulate and medial prefrontal cortex connectivity associated with comprehension and task focus while reading. Frontiers in human neuroscience. 2013;7. 10.3389/fnhum.2013.00734 PMC3825257 24282397

[31] Smallwood J, Nind L, O'Connor RC. When is your head at? An exploration of the factors associated with the temporal focus of the wandering mind. Conscious Cogn. 2009;18(1):118–25. Epub 2009/01/06. 10.1016/j.concog.2008.11.004 . 19121953

[32] Seli P, Carriere JS, Levene M, Smilek D. How few and far between? Examining the effects of probe rate on self-reported mind wandering. Frontiers in psychology. 2013;4. 10.3389/fpsyg.2013.00430 PMC3713396 23882239

[33] Ruby FJ, Smallwood J, Engen H, Singer T. How self-generated thought shapes mood—the relation between mind-wandering and mood depends on the socio-temporal content of thoughts. PloS one. 2013;8(10):e77554 Epub 2013/11/07. 10.1371/journal.pone.0077554 24194889 PMC3806791

[34] Ruby FJ, Smallwood J, Sackur J, Singer T. Is self-generated thought a means of social problem solving? Frontiers in psychology. 2013;4:962 Epub 2014/01/07. 10.3389/fpsyg.2013.00962 24391621 PMC3870294

[35] Engert V, Smallwood J, Singer T. Mind your thoughts: Associations between self-generated thoughts and stress-induced and baseline levels of cortisol and alpha-amylase. Biological psychology. 2014;103:283–91. 10.1016/j.biopsycho.2014.10.004 25457636

[36] Davey J, Cornelissen PL, Thompson HE, Sonkusare S, Hallam G, Smallwood J, et al Automatic and Controlled Semantic Retrieval: TMS Reveals Distinct Contributions of Posterior Middle Temporal Gyrus and Angular Gyrus. The Journal of Neuroscience. 2015;35(46):15230–9. 10.1523/JNEUROSCI.4705-14.2015 26586812 PMC4649000

[37] Jenkinson M, Smith S. A global optimisation method for robust affine registration of brain images. Medical image analysis. 2001;5(2):143–56. 11516708 10.1016/s1361-8415(01)00036-6

[38] Jenkinson M, Bannister P, Brady M, Smith S. Improved optimization for the robust and accurate linear registration and motion correction of brain images. Neuroimage. 2002;17(2):825–41. 12377157 10.1016/s1053-8119(02)91132-8

[39] Smith SM. Fast robust automated brain extraction. Human brain mapping. 2002;17(3):143–55. 12391568 10.1002/hbm.10062 PMC6871816

[40] Behzadi Y, Restom K, Liau J, Liu TT. A component based noise correction method (CompCor) for BOLD and perfusion based fMRI. Neuroimage. 2007;37(1):90–101. 17560126 10.1016/j.neuroimage.2007.04.042 PMC2214855

[41] Murphy K, Birn RM, Handwerker DA, Jones TB, Bandettini PA. The impact of global signal regression on resting state correlations: are anti-correlated networks introduced? Neuroimage. 2009;44(3):893–905. 10.1016/j.neuroimage.2008.09.036 18976716 PMC2750906

[42] Margulies DS, Vincent JL, Kelly C, Lohmann G, Uddin LQ, Biswal BB, et al Precuneus shares intrinsic functional architecture in humans and monkeys. Proceedings of the National Academy of Sciences. 2009;106(47):20069–74. 10.1073/pnas.0905314106 PMC2775700 19903877

[43] Braga RM, Sharp DJ, Leeson C, Wise RJ, Leech R. Echoes of the brain within default mode, association, and heteromodal cortices. The Journal of Neuroscience. 2013;33(35):14031–9. 10.1523/JNEUROSCI.0570-13.2013 23986239 PMC3810536

[44] Leech R, Braga R, Sharp DJ. Echoes of the brain within the posterior cingulate cortex. The Journal of Neuroscience. 2012;32(1):215–22. 10.1523/JNEUROSCI.3689-11.2012 22219283 PMC6621313

[45] Behrens TE, Sporns O. Human connectomics. Current opinion in neurobiology. 2012;22(1):144–53. 10.1016/j.conb.2011.08.005 21908183 PMC3294015

[46] van den Heuvel MP, Sporns O. Rich-club organization of the human connectome. The Journal of Neuroscience. 2011;31(44):15775–86. 10.1523/JNEUROSCI.3539-11.2011 22049421 PMC6623027

[47] Bzdok D, Heeger A, Langner R, Laird AR, Fox PT, Palomero-Gallagher N, et al Subspecialization in the human posterior medial cortex. NeuroImage. 2015;106:55–71. 10.1016/j.neuroimage.2014.11.009 25462801 PMC4780672

[48] Beaty RE, Benedek M, Silvia PJ, Schacter DL. Creative cognition and brain network dynamics. Trends in cognitive sciences. 2016;20(2):87–95. 10.1016/j.tics.2015.10.004 26553223 PMC4724474

[49] Brewer JA, Worhunsky PD, Gray JR, Tang Y-Y, Weber J, Kober H. Meditation experience is associated with differences in default mode network activity and connectivity. Proceedings of the National Academy of Sciences. 2011;108(50):20254–9. 10.1073/pnas.1112029108 PMC3250176 22114193

[50] Pagnoni G. Dynamical properties of BOLD activity from the ventral posteromedial cortex associated with meditation and attentional skills. The Journal of Neuroscience. 2012;32(15):5242–9. 10.1523/JNEUROSCI.4135-11.2012 22496570 PMC3362741

[51] Baird B, Smallwood J, Schooler JW. Back to the future: Autobiographical planning and the functionality of mind-wandering. Conscious Cogn. 2011 Epub 2011/09/16. 10.1016/j.concog.2011.08.007 . 21917482

[52] Song X, Wang X. Mind wandering in Chinese daily lives—an experience sampling study. PloS one. 2012;7(9):e44423 Epub 2012/09/08. 10.1371/journal.pone.0044423 22957071 PMC3434139

[53] Iijima Y, Tanno Y. [The effect of cognitive load on the temporal focus of mind wandering]. Shinrigaku kenkyu: The Japanese journal of psychology. 2012;83(3):232–6. 23012825 10.4992/jjpsy.83.232

[54] Smallwood J, Fitzgerald A, Miles LK, Phillips LH. Shifting moods, wandering minds: negative moods lead the mind to wander. Emotion. 2009;9(2):271 10.1037/a0014855 19348539

[55] Poerio GL, Totterdell P, Miles E. Mind-wandering and negative mood: Does one thing really lead to another? Conscious Cogn. 2013;22(4):1412–21. 10.1016/j.concog.2013.09.012 24149091

[56] Hassabis D, Maguire EA. The construction system of the brain. Philosophical Transactions of the Royal Society B: Biological Sciences. 2009;364(1521):1263–71. 10.1098/rstb.2008.0296 19528007 PMC2666702

[57] Baird B, Smallwood J, Lutz A, Schooler JW. The Decoupled Mind: Mind-wandering Disrupts Cortical Phase-locking to Perceptual Events. 2014. 10.1162/jocn_a_00656 24742189

[58] Smallwood J, Beach E, Schooler JW, Handy TC. Going AWOL in the brain: mind wandering reduces cortical analysis of external events. Journal of cognitive neuroscience. 2008;20(3):458–69. Epub 2007/11/17. 10.1162/jocn.2008.20037 . 18004943

[59] Smallwood J. Distinguishing how from why the mind wanders: a process-occurence framework for self generated thought. Psychological bulletin. 2013. 10.1037/a0030010 23607430

[60] Smallwood J. Searching for the Elements of Thought: Reply to Franklin, Mrazek, Broadway, and Schooler (2013). Psychological bulletin. 2013;139(3):542–7. 10.1037/A0031019 . 23607432

[61] Bzdok D, Langner R, Schilbach L, Jakobs O, Roski C, Caspers S, et al Characterization of the temporo-parietal junction by combining data-driven parcellation, complementary connectivity analyses, and functional decoding. Neuroimage. 2013;81:381–92. 10.1016/j.neuroimage.2013.05.046 23689016 PMC4791053

[62] Moran JM, Macrae CN, Heatherton TF, Wyland C, Kelley WM. Neuroanatomical evidence for distinct cognitive and affective components of self. Journal of cognitive neuroscience. 2006;18(9):1586–94. 16989558 10.1162/jocn.2006.18.9.1586

[63] Baird B, Cieslak M, Smallwood J, Grafton ST, Schooler JW. Regional White Matter Variation Associated with Domain-specific Metacognitive Accuracy. Journal of cognitive neuroscience. 2015. 10.1162/jocn_a_00741 25313660

[64] Fleming SM, Huijgen J, Dolan RJ. Prefrontal contributions to metacognition in perceptual decision making. The Journal of Neuroscience. 2012;32(18):6117–25. 10.1523/JNEUROSCI.6489-11.2012 22553018 PMC3359781

[65] Fleming SM, Weil RS, Nagy Z, Dolan RJ, Rees G. Relating introspective accuracy to individual differences in brain structure. Science. 2010;329(5998):1541–3. 10.1126/science.1191883 20847276 PMC3173849

